# ^18^F-NaF and ^18^F-FDG as molecular probes in the evaluation of atherosclerosis

**DOI:** 10.1007/s00259-018-4078-0

**Published:** 2018-07-06

**Authors:** Mikaela L. McKenney-Drake, Mateen C. Moghbel, Koosha Paydary, Mouhamad Alloosh, Sina Houshmand, Sharon Moe, Ali Salavati, Jeffrey M. Sturek, Paul R. Territo, Connie Weaver, Thomas J. Werner, Poul Flemming Høilund-Carlsen, Michael Sturek, Abass Alavi

**Affiliations:** 1 Department of Health Sciences, Butler University, Indianapolis, IN USA; 2 Department of Radiology, Stanford University Medical Center, Stanford, CA USA; 3 Department of Radiology, Hospital of the University of Pennsylvania, 3400 Spruce Street, Philadelphia, PA 19104 USA; 4 Department of Cellular & Integrative Physiology, Indiana University School of Medicine, Indianapolis, IN USA; 5 Department of Medicine, Indiana University School of Medicine and Roudebush Veterans Administration Medical Center, Indianapolis, IN USA; 6 Department of Radiology, University of Minnesota, Minneapolis, MN USA; 7 Department of Medicine, University of Virginia, Charlottesville, VA USA; 8 Department of Radiology & Imaging Sciences, Indiana University School of Medicine, Indianapolis, IN USA; 9 Department of Nutrition Science, Purdue University, West Lafayette, IN USA; 10 Department of Nuclear Medicine, Odense University Hospital, Odense, Denmark

**Keywords:** Atherosclerosis, ^18^F-FDG, ^18^F-NaF, Calcification, Global assessment of cardiac disease, Cardiovascular disease quantification

## Abstract

The early detection of atherosclerotic disease is vital to the effective prevention and management of life-threatening cardiovascular events such as myocardial infarctions and cerebrovascular accidents. Given the potential for positron emission tomography (PET) to visualize atherosclerosis earlier in the disease process than anatomic imaging modalities such as computed tomography (CT), this application of PET imaging has been the focus of intense scientific inquiry. Although ^18^F-FDG has historically been the most widely studied PET radiotracer in this domain, there is a growing body of evidence that ^18^F-NaF holds significant diagnostic and prognostic value as well. In this article, we review the existing literature on the application of ^18^F-FDG and ^18^F-NaF as PET probes in atherosclerosis and present the findings of original animal and human studies that have examined how well ^18^F-NaF uptake correlates with vascular calcification and cardiovascular risk.

## Introduction

As a major contributor to many life-threatening and debilitating diseases, atherosclerosis is among the foremost causes of morbidity and mortality throughout the world [1]. It is directly responsible for the majority of ischemic cardiovascular and cerebrovascular events, which claim the lives of 7.0 and 2.8 million people every year, respectively [2]. Moreover, it is an underlying cause of peripheral vascular disease, which affects 202 million individuals globally [3]. The economic toll of atherosclerosis is equally staggering; in the US alone, heart disease and stroke are estimated to cost over $240 billion annually [4].

**Fig. 1:**
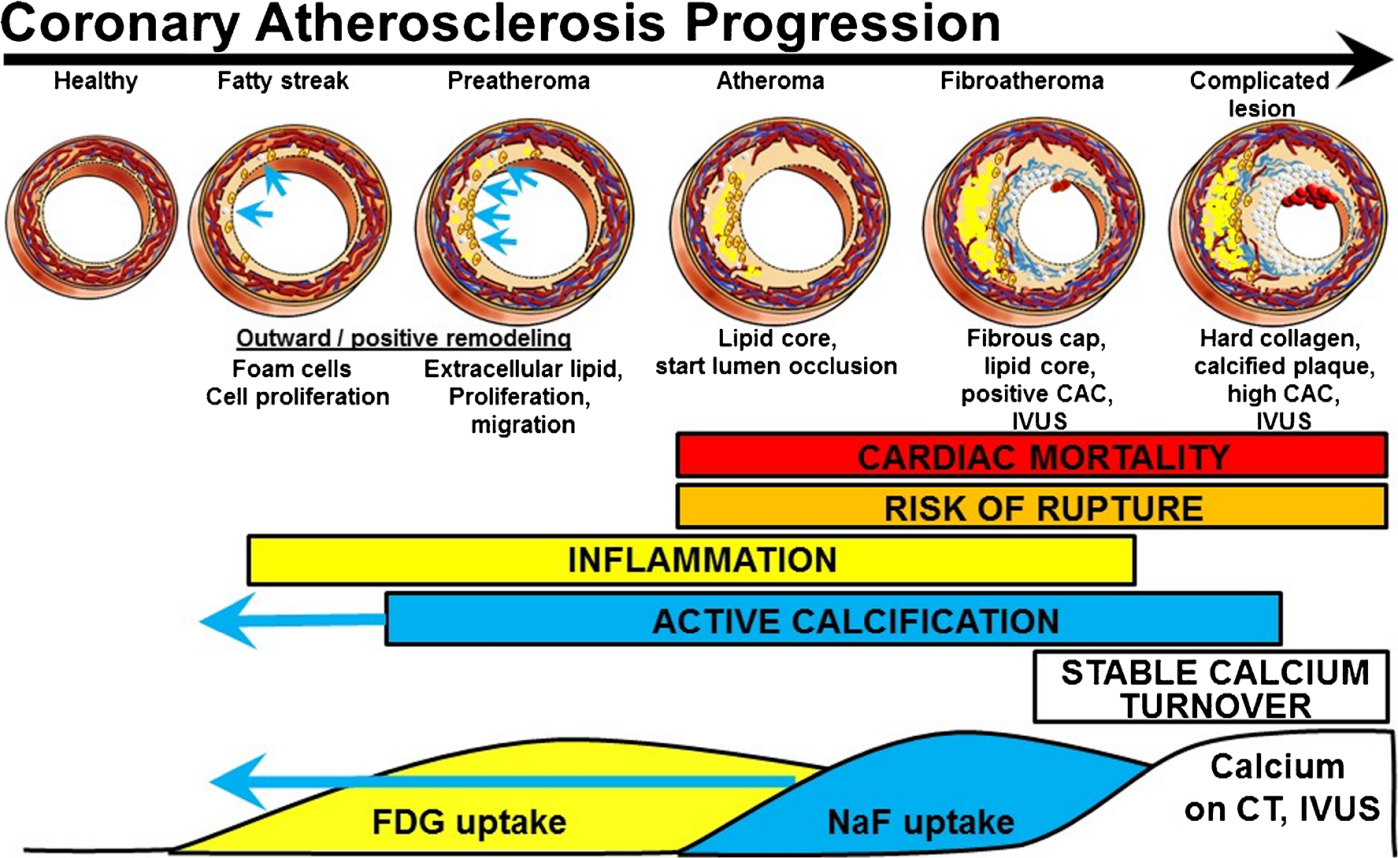
Progression from healthy arteries to complicated lesions. FDG and NaF uptake have long been known to precede vascular calcification evident on CT and intravascular ultrasonography (IVUS) [5, 6]. The paradigm shift is the stronger predictive power of NaF uptake and the occurrence of active calcification measured by ^18^F-NaF uptake in early coronary fatty streaks and preatheroma (*CAC* coronary artery calcium)

Risk factors such as hypertension, hypercholesterolemia, diabetes, and smoking put individuals at risk of developing atherosclerosis by damaging the vascular endothelium and setting off a cascade of proinflammatory responses as shown schematically in Fig. 1 [7]. In the initial stages, vascular permeability is increased, allowing for infiltration and retention of low-density lipoprotein cholesterol (LDL), and peripheral monocytes are drawn into the intimal layer and transformed into macrophages. As these LDLs are oxidized, they are ingested by macrophages, forming foam cells that subsequently undergo apoptosis and necrosis. The resulting breakdown products, including oxidized lipids and cellular debris, make up a highly thrombogenic lipid core covered by a collagen-rich fibrous cap. These plaques can expand with further accumulation of lipids and eventually rupture with the weakening of the fibrous cap, causing life-threatening events such as myocardial infarction and ischemic stroke.

The inflammatory process of plaque formation triggers another chain of events that gives rise to the calcification of the vascular wall [8]. In the early phases of this progression, cytokines released by inflammatory cells, such as TNF-α and IGF-1, induce the formation of lipid-laden plaques as well as the osteogenic transformation of the surrounding vascular smooth muscle cells. These changes create a positive feedback loop that causes further osteogenic differentiation and gives rise to microcalcifications that coalesce and ultimately pervade the atherosclerotic plaque, intimal layer, and medial layer [9]. Calcifications under 50 μm in size are generally considered microcalcifications, which are a marker of cell death and inflammation and carry an increased risk of plaque rupture and associated complications. Macrocalcifications, on the other hand, measure greater than 50 μm and may actually impart plaque stability [10]. Clinically, plaques at this stage of calcification may be irreversible. A review of randomized controlled clinical trials has not shown that therapeutic interventions (e.g. statins) slow the progression of coronary artery calcification [5]. Further, high-intensity statin therapy has been found to increase calcification [11]. The mechanism proposed was substantial delipidation and increased vascular smooth muscle calcification.

Given the enormous human and economic cost of atherosclerosis and its downstream effects, early and effective detection—particularly of plaques vulnerable to rupture—is paramount. Assessment of Framingham risk factors such as dyslipidemia, hypertension, and diabetes can identify those at risk of developing atherosclerotic disease, but cannot shed light on the extent or vulnerability of existing plaques. Clinical evaluations performed in symptomatic patients, such as the ankle-brachial index and cardiac stress tests, offer some insight into the presence of intraluminal stenosis, but the degree of stenosis alone has not been shown to be predictive of plaque rupture [7, 12]. The most reliable means of risk stratification therefore remains radiologic examination, which can visualize not only luminal stenosis but also plaque morphology. The plaque composition most strongly associated with rupture includes a thin fibrous cap, a lipid-rich necrotic core, neovascularization, intraplaque hemorrhage, and microcalcifications [13].

The anatomic imaging modalities that have historically been used in the assessment of atherosclerosis have tended to focus on noncompositional parameters. Conventional ultrasonography and angiography techniques can be used to examine the extent of luminal stenosis, while cardiac CT can be used to produce calcium scores to quantify the calcification of the coronary vessels. However, other modalities, including multidetector CT coronary angiography, MRI, intravascular ultrasonography (IVUS), and optical coherence tomography, have begun to be implemented with the specific aim of determining plaque composition [13, 14]. By virtue of being anatomic in nature, these modalities are best suited to illustrating late-stage structural changes or calcifications within the vessel walls, and lack the sensitivity and chemical specificity for early detection.

Unlike their anatomic counterparts, molecular imaging modalities are capable of detecting microscopic processes such as inflammation and microcalcification. These chemical composition changes occur early in the disease process and precede the aforementioned morphologic developments (Fig. 1). Foremost among these molecular techniques is positron emission tomography (PET) using the radiotracer ^18^F-fluorodeoxyglucose (^18^F-FDG), a radiolabeled glucose analog that serves as a marker of metabolic activity and, by extension, inflammation. By contrast, ^18^F-sodium fluoride (^18^F-NaF) is a specific marker of bone mineralization that has traditionally been used in diagnosing metastatic bone cancer, but has recently been applied to vascular calcification. The aim of this review was to examine the available literature on ^18^F-NaF PET as a diagnostic and prognostic tool in comparison with the more established method of ^18^F-FDG PET.

## ^18^F-FDG in the detection of atherosclerosis

^18^F-FDG, the most widely utilized PET radiotracer by far, was among the first molecular probes used for the assessment of atherosclerosis and is perhaps the best studied to date. Early studies showed a correlation between ^18^F-FDG activity within the vasculature and atherosclerotic and cardiovascular risk factors, promoting its utility as a diagnostic agent [15–18]. Further investigation revealed more direct evidence of a link between ^18^F-FDG uptake and atherosclerotic disease. Studies in patients with a history of cerebrovascular accidents showed elevated ^18^F-FDG activity in 58–85% of carotid lesions [19, 20]. Tawakol et al. performed PET scans in patients with severe carotid stenosis prior to endarterectomy, and found a significant correlation between ^18^F-FDG uptake in carotid plaques and macrophage staining on corresponding pathology specimens [21]. In a similar study, Rudd et al. used autoradiography to demonstrate that ^18^F-FDG-avid lesions in patients with symptomatic carotid stenosis line up with macrophage-rich areas of plaque in endarterectomy specimens [22]. The authors also found that ^18^F-FDG uptake was 27% higher in symptomatic carotid lesions than in contralateral asymptomatic lesions [22].

Other studies have provided some evidence that ^18^F-FDG PET imaging can be used to evaluate plaque vulnerability and the risk of associated ischemic events. Figueroa et al. found that plaques with high-risk morphologic characteristics on CT and histopathology have significantly higher ^18^F-FDG avidity [23]. Patients with increased arterial ^18^F-FDG uptake have been found to be significantly more likely to go on to suffer an ischemic cardiovascular event [24] or a cerebrovascular event [25].

The role of ^18^F-FDG PET in monitoring the effects of various treatments for atherosclerotic disease has also been investigated. In a study of 43 patients undergoing ^18^F-FDG PET for cancer screening, Tahara et al. randomized subjects to undergo either pharmacologic intervention with simvastatin or dietary modification alone, and found that only the treatment group showed a significant decrease in plaque SUV [26]. Ogawa et al. randomized hyperlipidemic rabbits to receive either the antioxidant probucol or no intervention at all, and found that the SUV of the aorta decreased significantly in the former group and increased gradually in the latter group [27]. They also found that the animals in the treatment arm demonstrated significantly lower macrophage infiltration of the aorta on histology [27]. In a similar study using a rabbit model, Worthley et al. found that aortic ^18^F-FDG uptake significantly increased in animals in which an atherogenic diet was continued and decreased in those in which a normal diet was restored [28].

Although these findings strongly suggest that ^18^F-FDG activity is associated with the atherosclerotic disease process, this link is distinct from the structural changes captured by anatomic imaging modalities. Across multiple studies, ^18^F-FDG avidity has been shown to be highly discordant with CT findings. In a study of 85 patients, Tatsumi et al. found no correlation between ^18^F-FDG uptake and CT calcium score [29]. Similarly, studies comparing sites of hypermetabolism on PET with those of hyperdensity on CT have shown vastly incongruent results. Ben-Haim et al. and Dunphy et al., in studies including 122 and 78 patients, found agreement in only 7% and 2% of cases, respectively [30, 31]. Patients with high ^18^F-FDG uptake were generally found to be younger than those with extensive calcification, suggesting that high ^18^F-FDG uptake represents an earlier stage in the disease process.

It has been postulated that uptake of ^18^F-FDG is representative of the inflammatory activity of activated macrophages infiltrating the fibrous cap, which predisposes plaques to rupture [32]. However, this has been a point of contention in the literature. A study in eight patients with carotid atherosclerosis showed heavy macrophage infiltration in plaques with ^18^F-FDG avidity [22], while another study in 21 patients with peripheral artery disease showed no significant correlation between ^18^F-FDG activity and levels of CD68, a measure of macrophage content [33].

Perhaps the most significant limitation of ^18^F-FDG as a probe for atherosclerosis is its intense uptake by myocardial tissue. The spillover from this physiologic activity obscures pathologic inflammation from atherosclerotic plaques, greatly limiting the utility of the modality in assessing the coronary vasculature. A method of mitigating this limitation is to suppress myocardial glucose metabolism by requiring the patient to fast or consume a high-fat, low-carbohydrate diet prior to image acquisition [34–36]. Although these protocols are in clinical use for mediastinal pathologies such as sarcoidosis, they have not been as widely studied or implemented in coronary atherosclerosis. As a result, ^18^F-FDG is most commonly used to evaluate atherosclerosis within the carotid arteries, the aorta, and its larger offshoots. However, even during delayed imaging of these structures, the contrast resolution is limited by the retention of radiotracer in the surrounding tissues, blood pool, and vessel wall. Another limitation, which applies to ^18^F-FDG as well as ^18^F-NaF, is the spatial resolution of PET; visualization of microcalcifications in the millimeter range may be constrained by the physical limits of the modality [21, 37].

## ^18^F-NaF in the detection of atherosclerosis

Although its application to vascular imaging is relatively recent, ^18^F-NaF has shown considerable promise in allowing the evaluation of patients at risk of atherosclerosis. It differs from ^18^F-FDG PET in its molecular binding characteristics and, thus, the manner in which it illustrates disease burden. Whereas ^18^F-FDG is taken up by metabolically active cells and is considered a surrogate for inflammation, ^18^F-NaF is incorporated into areas of calcium deposition by exchanging the hydroxyl ions of hydroxyapatite crystals with radiolabeled fluoride to form fluorapatite [38]. Consequently, ^18^F-NaF PET scans are not affected by the major limitation of myocardial uptake and can be used to assess the coronary arteries in addition to the peripheral vasculature. The uptake of ^18^F-NaF in bone is orders of magnitude greater than in the vasculature, and thus it is vital to focus imaging on the vasculature to minimize background bone uptake of the tracer.

Several studies have shown that vascular uptake of ^18^F-NaF is not only correlated with advancing age, but also with risk factors for atherosclerotic and cardiovascular disease [39–43]. This was the earliest evidence that the modality could be diagnostic for atherosclerosis and spurred a series of confirmatory studies. Among these was a prospective trial performed by Joshi et al. in 40 patients undergoing ^18^F-NaF PET/CT after myocardial infarction [44]. Uptake was highest in the culprit plaque in 93% of patients. Moreover, plaques with high ^18^F-NaF activity were significantly more likely to demonstrate high-risk morphologic features—namely, positive remodeling, microcalcification, and necrosis of the lipid core—on ultrasonography. It is plausible that the observed increase in NaF uptake in culprit lesions is attributable to the permeability of ruptured plaques, but there is no evidence of this to our knowledge. The fact that patients were imaged after myocardial infarction limits the ability of the study to inform risk assessment in patients prior to plaque rupture. Longitudinal NaF studies in the same subjects in the absence and presence of myocardial infarction/stenting are needed.

Recent research effort has sought to validate ^18^F-NaF PET in comparison with existing diagnostic tools. More specifically, the distribution of vascular uptake has been compared with that of calcified plaques visualized on CT. In a retrospective study in 61 patients, Li et al. found a significant correlation between ^18^F-NaF uptake and calcification on CT in the coronary arteries, but not in the aorta [45]. Derlin et al. performed a similar study in 75 patients, most of whom exhibited multiple sites of ^18^F-NaF uptake and arterial calcification on CT. Notably, the vast majority (88%) of sites with ^18^F-NaF activity on PET were colocalized with calcifications seen on CT, but far fewer sites of calcification (12%) showed elevated ^18^F-NaF uptake [46]. These results suggest that ^18^F-NaF activity can indicate the presence of microcalcifications before they are large enough to be visualized on CT. Moreover, the lack of ^18^F-NaF uptake at sites of macrocalcification discernible by anatomic imaging techniques suggests that these plaques are no longer undergoing active calcium deposition.

The clinical application of ^18^F-NaF PET in atherosclerosis requires validation in large prospective studies, as much of the data that has appeared in the literature thus far has been derived from single center retrospective studies with limited sample sizes. Additional prospective studies are especially vital to determining the modality’s prognostic value, which hinges on the ability to identify plaques at risk of rupture. The specificity of ^18^F-NaF for this application is also worthy of investigation; although not as intense as myocardial uptake of ^18^F-FDG, background uptake of ^18^F-NaF may be sufficient in some patients to preclude differentiation between vulnerable and stable plaques.

## ^18^F-NaF as a marker of cardiovascular microcalcification in animal studies

The potential of ^18^F-NaF PET for the early detection of atherosclerosis has been illustrated most vividly in animal studies. McKenney-Drake et al. and Salavati et al. used the Ossabaw miniature swine model of metabolic syndrome (MetS) to demonstrate that ^18^F-NaF uptake in the coronary arteries precedes the emergence of macroscopic calcification on IVUS and CT scans [47–49].

To investigate NaF as a marker of early coronary artery calcium (CAC, microcalcification), a study was conducted in this preclinical model. The study included 13 lean (control) swine and 11 swine with MetS and early coronary artery disease (CAD). When fed a high-calorie atherogenic diet and forced to live a sedentary lifestyle, the Ossabaw swine developed progressive CAD from the stages of clinically insignificant fatty streaks through necrotic, flow-limiting lesions with macrocalcifications detectable on IVUS [50, 51]. CAC was measured using invasive methods (IVUS, histopathology) to compare with ^18^F-NaF uptake in the coronary arteries to test the hypothesis that ^18^F-NaF PET imaging can detect CAC earlier than the current gold standard noninvasive CT scanning [47–49]. Details related to the characterization of the Ossabaw swine have been previously reported [50].

**Fig. 2:**
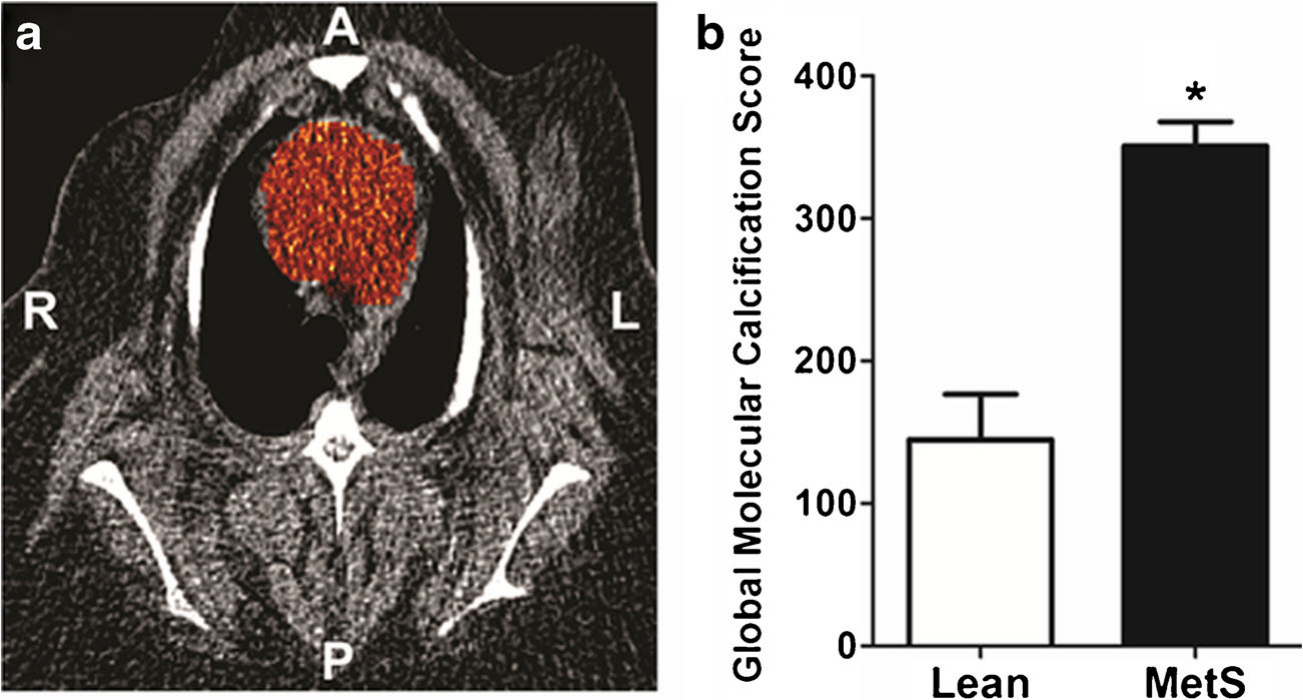
Coronary artery global molecular calcification score (GMCS) and percent injected dose per gram body weight ^18^F-NaF uptake. **a** A region of interest was drawn around the heart on each cardiac CT slice from which GMCS was calculated. **b** Pigs with metabolic syndrome (MetS; *n* = 11) had a GMCS almost 2.5-fold higher than lean pigs (*n* = 2; *p < 0.05)

**Fig. 3:**
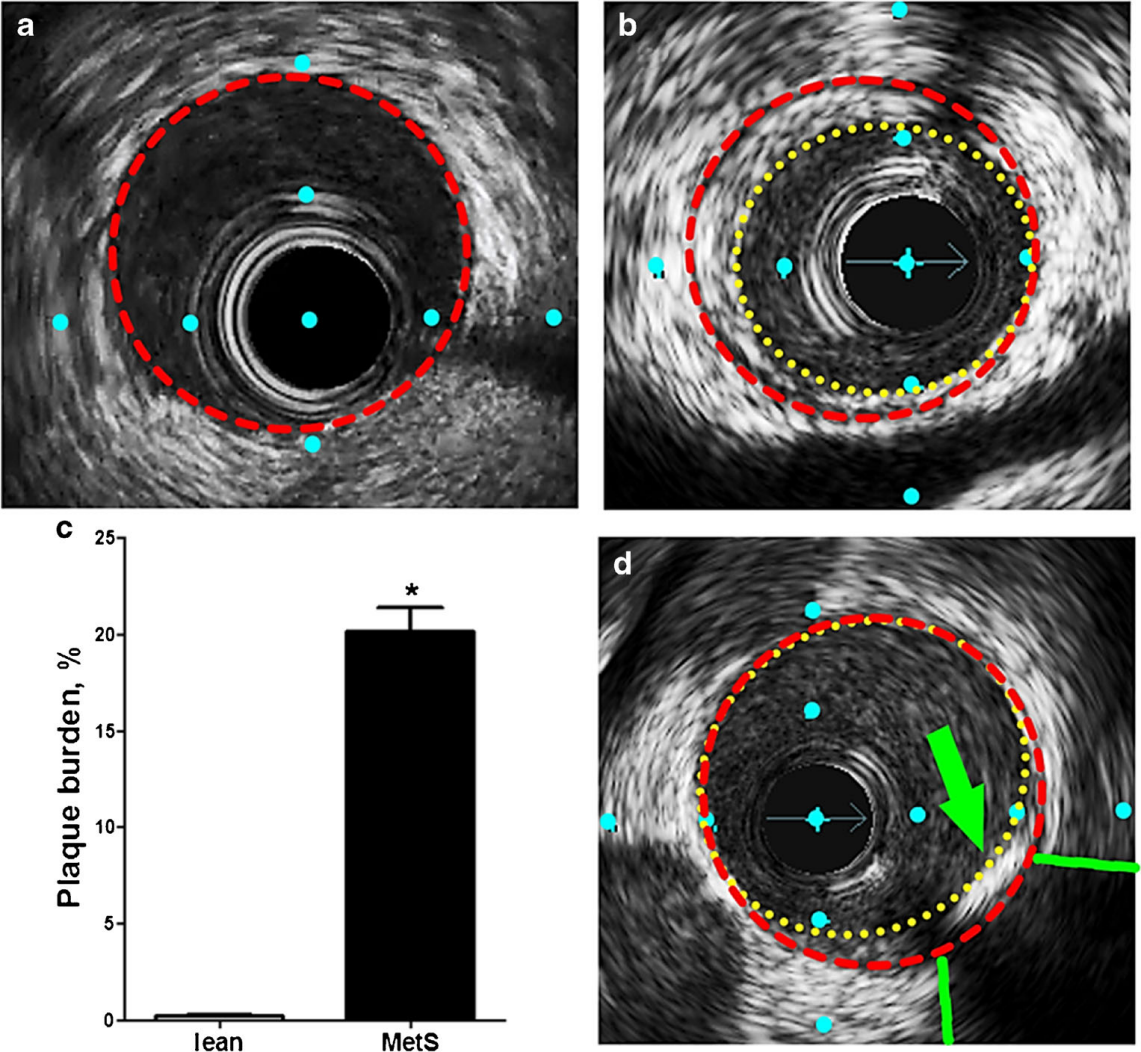
IVUS images and quantification of early-stage CAD (type I, II, and III lesions). **a**, **b**, **d** Cross-sectional view of a coronary artery in Ossabaw pigs. **a** A lean pig (vascular wall traced in *red*). **b** A pig with metabolic syndrome and CAD (initial lumen traced in *red*, actual lumen traced in *yellow*); percent plaque burden = (initial − actual)/initial) × 100. **c** Pigs with metabolic syndrome (MetS; *n* = 7) had significantly more extensive CAD than lean pigs (*n* = 2) as shown by plaque burden quantification in the proximal 15 mm of the right coronary artery (*p* < 0.05). **d** One pig showed evidence of focal calcification (*green arrow* lesion, *green lines* acoustic shadowing). Distance between blue dots is 1 mm

These studies relied on the Alavi-Carlsen global molecular calcium score (GMCS), a quantification system pioneered in an earlier ^18^F-NaF PET study by Beheshti et al. [43]. A measure of total cardiac uptake, the GMCS is calculated by summing the product of SUV and volume of the region of interest (ROI) on every slice within the borders of the heart [42, 43, 52] (Fig. 2a). All pigs, lean and MetS, showed no evidence of CAC on CT with an Agatston calcium score of 0. Simultaneous PET imaging showed that ^18^F-NaF uptake in the heart was almost 2.5-fold higher in MetS pigs than in lean pigs (GMCS 351 ± 17 vs. 145 ± 26, *p* < 0.05) [49] (Fig. 2b). Plaque burden was also measured using IVUS in these two groups of pigs. Images of the proximal 15 mm of the right coronary artery (RCA) showed no atherosclerotic plaque burden in lean pigs (Fig. 3a), in contrast to a plaque burden indicating the early stage of CAD in MetS pigs (Fig. 3b). MetS pigs had 100-fold greater plaque burden than lean pigs (0.2 ± 0% vs. 20 ± 1%, *p* < 0.05; Fig. 3c). Of the MetS pigs with CAD, two showed evidence of focal CAC on IVUS (Fig. 3d).

**Fig. 4:**
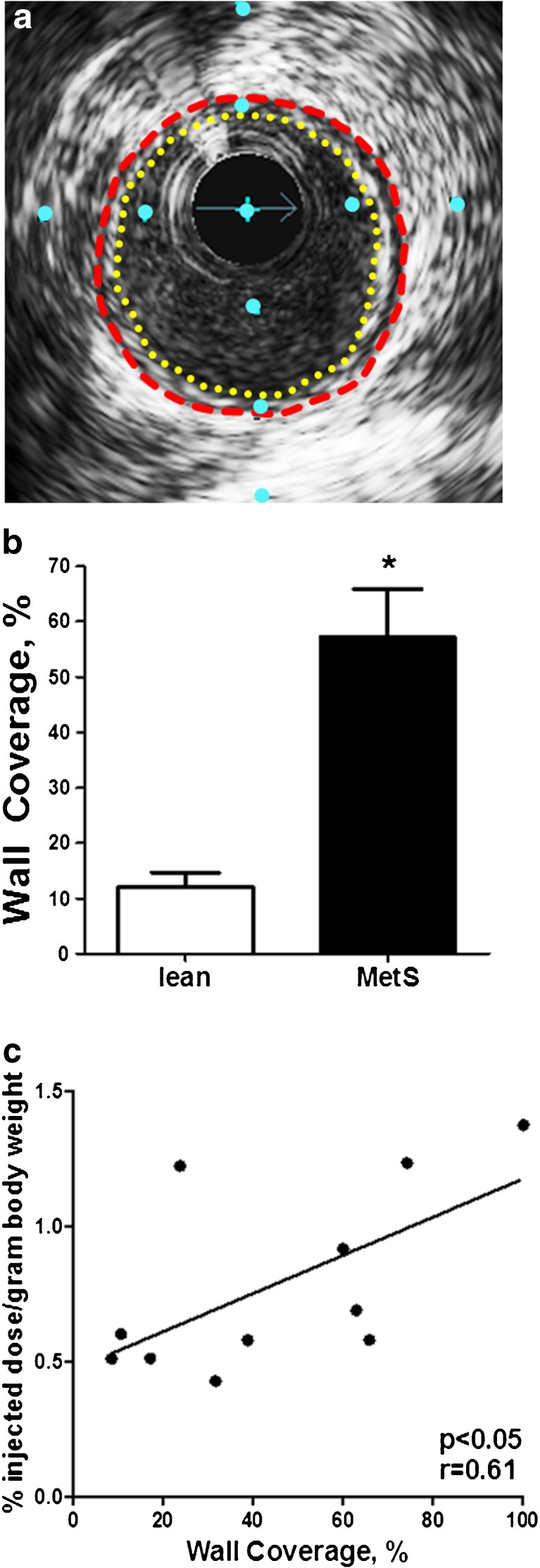
Early stage CAD was quantified as percent wall coverage using IVUS. **a** Cross-sectional image of a coronary artery in an Ossabaw pig with metabolic syndrome with 100% wall coverage (concentric fatty streak, intimal thickening) and a plaque burden of about 13%, which would not be clinically significant. **b** Pigs with metabolic syndrome (MetS; *n* = 8) had a percent wall coverage about fivefold greater than lean pigs (*n* = 3; *p < 0.05). **c** RCA intimal wall coverage is significantly correlated with ^18^F-NaF uptake (*p* < 0.05). Distance between blue dots is 1 mm

To better represent this early stage of CAD, another IVUS technique—percent wall coverage (PWC)—was used as a surrogate for atherosclerosis. The PWC of the proximal RCA was more variable than the percent plaque burden data. An example of early CAD (i.e., fatty streak, preatheroma, intimal thickening), with a low percent plaque burden (about 13%), is shown as concentric wall coverage (100%) in Fig. 4a. Strong evidence for PWC on IVUS as a quantitative measure of intimal thickening is the correlation between PWC and Verhoeff–van Gieson histologic staining (*r* = 0.93, *p* < 0.0001). As expected, in MetS pigs PWC was about fivefold greater than in lean pigs (57 ± 9% vs. 12 ± 3%, *p* < 0.05; Fig. 4b). Compared to ^18^F-NaF uptake in the RCA of each pig, PWC was significantly correlated with microcalcification activity measured on PET (*p* < 0.05, *r* = 0.61; Fig. 4c). These findings are reported by McKenney-Drake et al. [49] as separate values for the left anterior descending artery and the RCA.

**Fig. 5:**
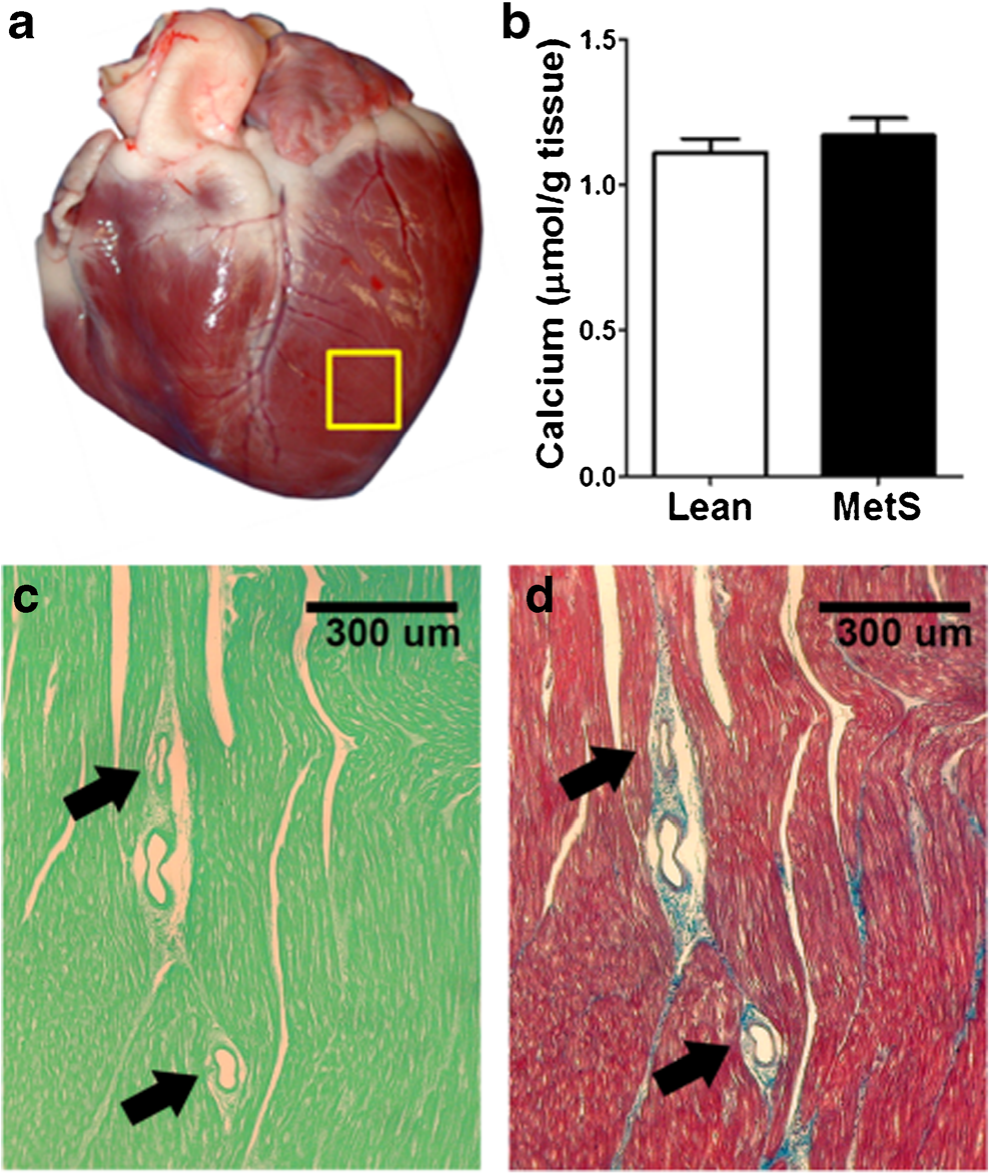
Calcium content of the left ventricle. **a** Specimen was collected from the left ventricle away from any conduit artery (*yellow box*). **b** There is no difference in left ventricle calcium content between lean pigs (*n* = 6) and MetS pigs (*n* = 11). **c**, **d** Histopathology of the left ventricle: **c** von Kossa mineral staining on fast green background (showing mineral as black sediment)) showed no evidence of myocardial or microvasculature calcification in either lean pigs or MetS pigs; **d** Masson’s trichrome staining shows healthy nonfibrotic myocardium (*arrows* microvessels)

Biochemical assessment revealed no significant difference in the calcium content of the left ventricle (Fig. 5a) between lean pigs (1.11 ± 0.05 μmol/g) and MetS pigs (1.17 ± 0.06 μmol/g, *p* = 0.24; Fig. 5b). Furthermore, histopathology did not reveal evidence of ventricular calcification (Fig. 5c, d). Taken together, these findings suggest that the measured ^18^F-NaF uptake did not come from the ventricular tissue of the heart.

This preclinical PET/CT study demonstrated that increased ^18^F-NaF uptake in coronary arteries is a biomarker for early CAC in pigs with CAD that lack frank evidence of calcification on IVUS and CT imaging. These findings imply that ^18^F-NaF binds to microcalcifications too small to be detected using anatomic/morphologic imaging modalities, an interpretation that is strengthened by histopathology data revealing sparse calcifications within the proximal region of the coronary artery. The histopathology findings did not significantly correlate with ^18^F-NaF uptake in the coronary arteries, suggesting that histologic assessment detects macroaggregates of hydroxyapatite, but not the microcalcifications visualized on ^18^F-NaF PET/CT [49]. The findings of this animal study and others that have used the same preclinical swine model of CAD [50, 51, 53, 54] hint at a role for ^18^F-NaF PET/CT in the early detection of atherosclerosis, but validation is required in human studies.

## ^18^F-NaF as a marker for cardiovascular microcalcification in human studies

**Fig. 6:**
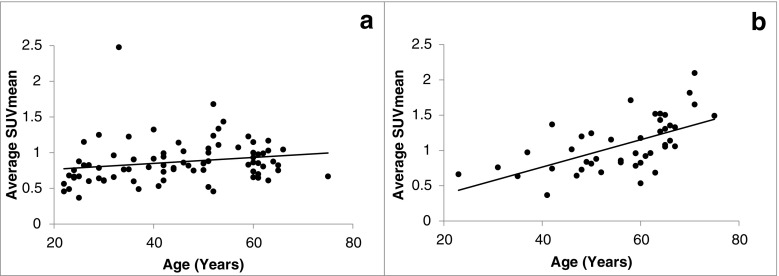
^18^F-NaF average SUVmean in the aortic arch wall in relation to age in healthy controls (**a**) and patients with cardiovascular risk factors (**b**). The average SUVmean (mean ± SD) in the aortic arch in healthy controls and patients were 0.87 ± 0.30 and 1.07 ± 0.37, respectively (*p* = 0.002). The Spearman correlation coefficients for the healthy controls and patients were 0.32 (*p* = 0.04) and 0.64 (*p* < 0.001), respectively

To further investigate the value of ^18^F-NaF PET/CT in the clinical evaluation of atherosclerosis, we analyzed 124 human subjects, including 80 healthy controls and 44 patients with chest pain syndromes. These subjects belonged to the Cardiovascular Molecular Calcification Assessed by ^18^F-NaF PET/CT (CAMONA) study in Odense, Denmark. All subjects underwent a 90-min ^18^F-NaF PET/CT scan and a 180-min ^18^F-FDG PET/CT scan under similar conditions. A whole-vessel analysis in axial sections was then performed for the three vascular segments of the thoracic aorta, including the ascending aorta, aortic arch and descending aorta. Measures of arterial microcalcification including average SUVmax, average SUVmean, and Alavi-Carlsen GMCS were calculated for each vascular segment as well as for the whole vessel using previously described methods. Vascular ^18^F-NaF uptake quantified as average SUVmax, average SUVmean, and GMCS was significantly higher in patients than in healthy controls. Of note, age was significantly correlated with average SUVmax, average SUVmean and GMCS in all vascular segments and the whole vessel, although the correlation coefficients were higher in patients with chest pain syndromes (Fig. 6). GMCS of the thoracic aorta was a stronger predictor of 10-year Framingham risk score (FRS) than average SUVmax and average SUVmean (adjusted *R*^2^ = 0.38, standardized *β* = 0.58, *p* < 0.001). Moreover, GMCS of the thoracic aorta was a borderline significant predictor of an unfavorable CVD risk profile as compared to average SUVmax and average SUVmean (odds ratio = 1.006, 95% CI = 1.000–1.013, *p* = 0.05). ^18^F-FDG uptake in the thoracic aorta quantified as average SUVmax and average SUVmean was positively correlated with age; however, the correlation was not significant in the healthy group.

**Fig. 7:**
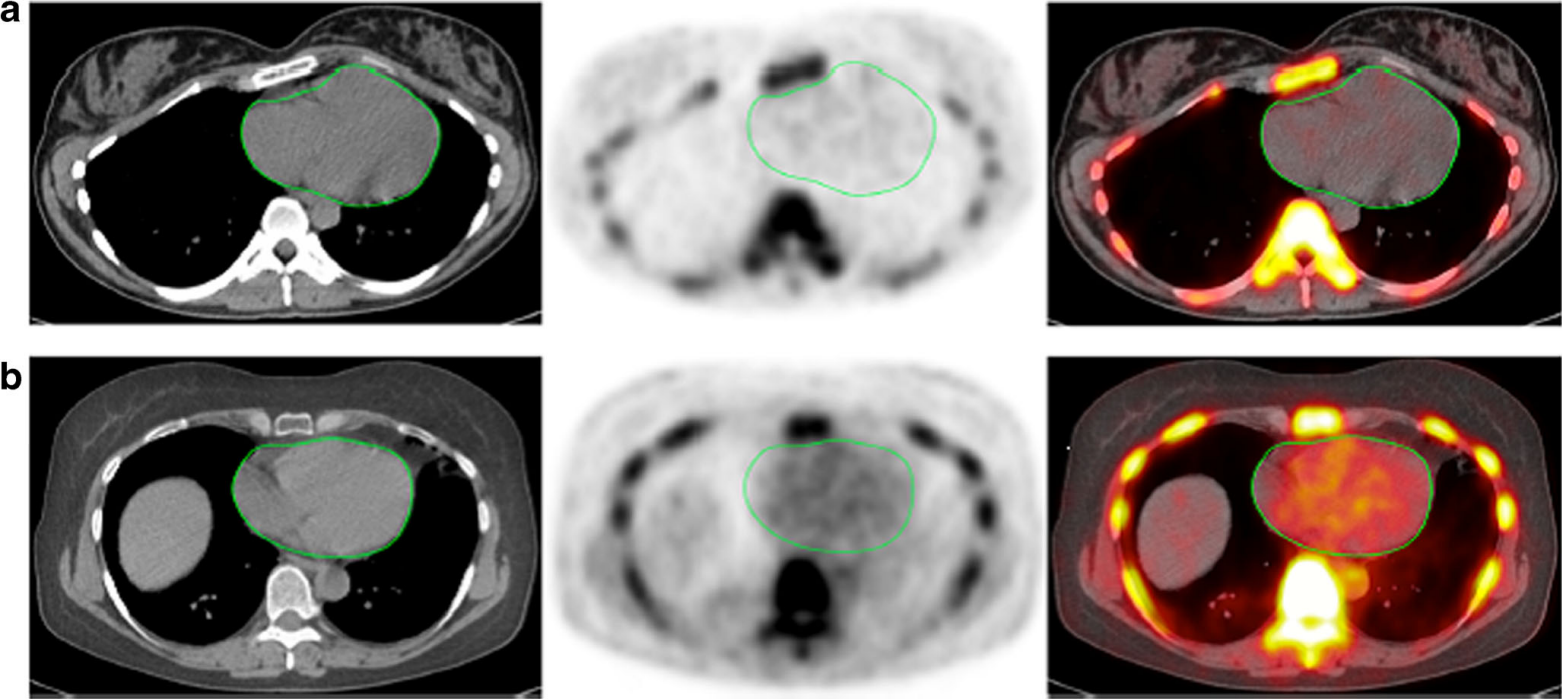
Transverse images (*left* CT, *middle* PET, *right* PET/CT) of the heart (*green circles*) in two clinically normal subjects (**a** 25 years old, **b** 61 years old). The global cardiac calcification scores were 12,492.44 in subject **a** and 18,424.70 in subject **b**. Normalizing the values to background NaF uptake increases the discrepancy between the subjects, resulting in 2.18 times the uptake in subject **b** than in subject **a**. Corresponding to the sites of NaF uptake in subject **b**, no structural calcification is seen on the corresponding CT scan and there is significant disparity between the PET and CT results. This is not an uncommon observation in this setting and clearly demonstrates the basis for assessment of cardiovascular calcification with these two different imaging modalities. While molecular imaging with NaF detects the earliest evidence for vascular calcification, evidence for calcification on CT largely reflects an end-stage disease process and therefore may be an irreversible pathologic state. Disparity between these two observations provides evidence for stage of calcification and has implications for the irreversibility of macrocalcification

We also assessed the extent of cardiac microcalcification in another subset of subjects from the CAMONA study that included 40 healthy subjects and 38 patients with chest pain syndromes. To quantify cardiac microcalcification, ellipsoid ROIs were manually drawn around cardiac ventricles by an experienced physician on axial PET/CT images starting from the base to the cardiac apex excluding the aortic valve (Fig. 7). Alavi-Carlsen GMCS was not correlated with age in healthy subjects (*r* = 0.11, *p* = 0.48), but was positively correlated with increasing age in patients with cardiovascular disease risk factors (*r* = 0.30, *p* = 0.04). These findings highly suggest that global assessment of cardiovascular microcalcification has potential applications for prompt risk stratification and therapeutic intervention in patients at high risk of cardiovascular disease. Given the evidence that ^18^F-NaF is able to detect arterial calcifications earlier than CT, this approach holds promise as a clinical alternative to the conventional CT-based calcium score.

## Comparing ^18^F-FDG and ^18^F-NaF as diagnostic and prognostic tools for atherosclerosis

Once the value of molecular imaging for evaluating patients at risk of atherosclerotic disease had been demonstrated, comparisons between available PET radiotracers became an area of scientific inquiry. Although numerous radiotracers have been investigated for this indication, including ^18^F-fluoromethylcholine [55, 56], a biomarker for structural wall alteration, ^18^F-FDG and ^18^F-NaF remain the most widely studied and compared.

Studies that have correlated ^18^F-FDG and ^18^F-NaF activity with clinical and pathologic measures of disease have consistently found that ^18^F-NaF is more strongly correlated. In a prospective study in 119 subjects, Dweck et al. found that, while ^18^F-NaF uptake in coronary arteries and the aorta was significantly correlated with 10-year FRS, ^18^F-FDG uptake was not [57]. Likewise, coronary ^18^F-NaF activity was significantly associated with increased rates of CAD and angina, as well as with histories of coronary revascularization and major adverse cardiac events, but ^18^F-FDG activity was not. In a prospective study of 139 subjects (89 healthy volunteers and 50 patients), Blomberg et al. similarly found that an increased risk of cardiovascular disease as estimated by FRS was associated with vascular calcification metabolism as measured by ^18^F-NaF, but was not associated with ^18^F-FDG uptake [42]. In a prospective study in 40 patients with prior myocardial infarction, Joshi et al. found that ^18^F-NaF uptake occurred very reliably in culprit plaques in the coronary arteries and histologically-confirmed sites of active calcification, macrophage infiltration, apoptosis, and necrosis [44]. No such relationship was observed for ^18^F-FDG. In both of these studies, the authors concluded that physiologic uptake of ^18^F-FDG by the myocardium obscured pathologic uptake in the coronary arteries, drastically limiting the ability to evaluate atherosclerotic burden using this radiotracer. Such comparisons were also extended to other cardiovascular conditions involving calcification and inflammation, producing similar results. In a study in 101 patients with aortic stenosis of varying severity and 20 controls, Dweck et al. found that uptake of both radiotracers was significantly higher in patients, but that ^18^F-NaF uptake was better correlated with the severity of valvular disease [58].

Studies comparing these molecular imaging probes with radiologic and serologic assessments of arterial calcification have also shown ^18^F-NaF to be better correlated. In a study in 45 patients, Derlin et al. found that ^18^F-NaF had far superior colocalization with CT calcifications than ^18^F-FDG (77.1% vs. 14.5%) [59], and also suggested that the specificity of ^18^F-NaF is superior to that of ^18^F-FDG, as the target-to-background uptake ratio of ^18^F-NaF (2.3) exceeded that of ^18^F-FDG. Two prospective studies by Dweck et al. confirmed that ^18^F-NaF uptake is significantly correlated with CT calcium scores, while ^18^F-FDG uptake is too intense in the myocardium to enable detection of coronary pathology [57, 60]. The more recent of the two studies [60] also compared the radiotracers with histologic measures of inflammation and calcification. Levels of CD68, the macrophage-associated inflammatory marker, were not associated with uptake of either agent. However, alkaline phosphatase and osteocalcin, surrogates for calcification, were found to be correlated with ^18^F-NaF alone.

Increasingly, there is evidence that molecular calcification may be the initial biologic process for development of atherosclerotic plaques in the arteries [61, 62]. This may improve our ability to detect this serious disorder in its earlier stages since ^18^F-NaF-based PET imaging is less prone to the complexities that are associated with PET imaging of this disorder.

## Conclusion

In this review, we have outlined evidence in the literature regarding the use of the molecular imaging probes ^18^F-NaF and ^18^F-FDG in detecting the chemical composition and vulnerability of atherosclerotic plaques. Despite possessing distinct binding properties that interrogate entirely different components of the disease process, these radiotracers were initially shown to be associated with many of the same clinical risk factors for atherosclerosis and cardiovascular disease [15–18, 39–41, 43]. Moreover, the uptake of both ^18^F-FDG and ^18^F-NaF was found to be increased not only in vulnerable plaques with high-risk morphologies, but also in ruptured plaques that cause myocardial infarctions and cerebrovascular accidents [25, 44].

However, the similarities ended when these PET probes were compared to anatomic imaging modalities, most notably CT calcium scores. While ^18^F-NaF correlated well with these conventional measures of atherosclerotic burden, ^18^F-FDG demonstrated no such relationship [29–31, 42, 46]. This observation speaks to the different mechanisms of action of these radiotracers. The role of ^18^F-NaF in illustrating the calcification that takes place in atherosclerotic disease is plainly evident in its correlation with CT calcium scores and colocalization with CT-detected macrocalcifications and pathologically confirmed microcalcifications. Thus, the data support the use of ^18^F-NaF PET as the best modality for the early detection of disease as it has been shown to pick up microcalcifications before they become visible on CT scans in humans [46] and even in fatty streaks in swine [36–38]. In contrast, ^18^F-FDG is not associated with these markers of calcification, but is linked to the inflammatory component of atherosclerosis, which is a critical risk factor for plaque rupture [32]. In addition, as a nonspecific glucose analog, ^18^F-FDG has the unique disadvantage of intense myocardial uptake, which obscures activity in the coronary arteries, and essentially restricts the applications of ^18^F-FDG to the peripheral vasculature [44, 57]. Largely because of this phenomenon, head-to-head studies comparing ^18^F-FDG and ^18^F-NaF have consistently shown that the latter is better correlated with clinical, pathologic, and radiologic measures of disease.

The relative specificity of ^18^F-NaF PET, as well as its comparative advantage in the early detection of microcalcifications (Fig. 1), position the modality well as a clinical tool for the diagnosis of atherosclerotic disease. The use of ^18^F-NaF PET could allow earlier intervention and improved outcomes, and studies combining this technique with CT calcium score or MRI may offer further sensitivity and specificity. A few early studies have already begun to demonstrate the utility of hybrid imaging for this indication [63, 64]. Given the promise and potential it has shown thus far, it appears that molecular imaging can, with continued validation, take hold in the clinical setting and improve our diagnosis and management of a disease that underlies some of the most common causes of morbidity and mortality throughout the world.

In two recent editorials, we have discussed current limitations of PET in assessing cardiovascular disorders and the suboptimal methodologies that are being employed in this structurally and biologically complicated domain [65, 66]. In particular, we have emphasized the challenges that are faced in overcoming image degradation due to cardiac and respiratory motion. We also have proposed global disease assessment in the coronary and major arteries to overcome these obstacles for accurate quantification of molecular abnormalities that are associated with atherosclerotic plaques.
